# Digital therapeutics for mental health: Is attrition the Achilles heel?

**DOI:** 10.3389/fpsyt.2022.900615

**Published:** 2022-08-05

**Authors:** Adaora Nwosu, Samantha Boardman, Mustafa M. Husain, P. Murali Doraiswamy

**Affiliations:** ^1^Department of Psychiatry and Behavioral Sciences, Duke University School of Medicine, Durham, NC, United States; ^2^Department of Psychiatry, Weill Cornell Medical College, New York, NY, United States; ^3^Departments of Psychiatry, Neurology and Biomedical Engineering, Southwestern Medical Center, The University of Texas, Austin, TX, United States; ^4^Duke Institute for Brain Sciences, Duke University, Durham, NC, United States

**Keywords:** ADHD, depression, anxiety, Alzheimer's disease, PTSD, autism, schizophrenia, addiction

## Introduction

Large unmet needs in mental health combined with the stress caused by pandemic mitigation measures have accelerated the use of digital mental health apps and software-based solutions (1). Global investor funding for virtual behavioral services and mental health apps in 2021 exceeded $5.5 billion, a 139% jump from 2020, according to CB Insights (2). While there are thousands of apps claiming to improve various aspects of mental wellbeing, many of them have never gone through clinical trials or regulatory scrutiny. The term “digital therapeutic” is used in the literature to distinguish high quality evidence-based software programs from wellness apps (3). Regulators use the term “software as a medical device” (SaMD) or “software in a medical device” (SiMD) to refer to software that functions as a medical device and is promoted to treat a specific condition. When a SaMD or SiMD is deployed on a phone it is referred to as a mobile medical app (MMA) (4, 5). The International Medical Device Regulators Forum (IMDRF), a voluntary group of medical device regulators from around the world has developed detailed guidance on definitions, framework for risk categorization, quality management, and the clinical evaluation of such devices (6–8). Non-traditional approaches, outside of RCTs, to evaluate efficacy for such tools has also been discussed elsewhere (9).

To date, only a few clinically tested software devices have been authorized by the U.S. Food and Drug Administration for treating specific mental health disorders (excluding devices marketed under pandemic-related emergency use authorization). These include reSET for substance abuse disorder (10), reSET-O for opioid use disorder (11), Somryst for chronic insomnia (12, 13) and EndeavorRx for pediatric attention deficit hyperactivity disorder (14, 15). SaMDs and MMAs for treating mild cognitive impairment, Alzheimer's disease, schizophrenia, autism, depression, social anxiety disorder, phobias and PTSD are in clinical trials (1, 3, 5, 16, 17) and may also come to market soon. The state of efficacy for non-regulated, wellness apps (e.g., for mindfulness or stress management) is beyond the scope of this article, and readers are referred elsewhere for information on these apps (16).

## High attrition and low engagement

While digital therapeutics and apps undoubtedly hold promise, relatively little attention has focused on attrition rates. Even effective apps will have limited impact if they are not highly engaging and result in high attrition (18, 19). Attrition, the loss of a randomized subject(s) from a study sample, is a very common issue in clinical trials and results from several causes such as refusal to participate after randomization, an early dropout from the study, and loss of subject's study data. Attrition can substantially bias estimates of efficacy and reduce generalizability (20). Traditionally, regulatory trials of psychopharmacological agents have used the last observation carried forward (LOCF) statistical method to accommodate attrition–but this has been increasingly replaced with mixed-effects models, and pattern-mixture and propensity adjustments (20). Compliance in trials of psychopharmacological agents is traditionally measured *via* pill counts. However, in virtual platform trials of digital therapeutics, compliance cannot simply be measured by the number of times a subject logs on to an app and it is important to also measure and report how engaged users were with the app (21). Currently, there is no standard way to define what constitutes meaningful engagement and how to compare engagement across different digital therapeutic devices (21). There is also no consensus as to how to deal with users who are non-engaged but stay in the study.

As patients typically use apps on their own time, they must be intrinsically motivated to do so and must perceive the benefits from the app as meaningful (18). Such intrinsic motivation may be low for psychiatric patients with depression, anhedonia, or cognitive difficulties. For example, in one study of internet-based cognitive behavioral therapy for depression, the highest engagers comprised just 10.6% of the sample (22). This is further highlighted by a 2020 meta-analysis of 18 randomized trials of (non-FDA cleared) mobile apps for treating depression (trial duration ranging from 10 days to 6 months), in which the pooled dropout rate was 26.2% and rose to 47.8% when adjusted for publication bias (19). The authors concluded that this raises concern over whether efficacy was overstated in these studies. Real-world attrition rates for non-FDA cleared mental wellness apps are not readily available for direct comparison. But one study of 93 non-FDA approved Android apps (median installs 100,000), targeting mental wellbeing, found the medians of app 15 and 30-day retention rates were very low at 3.9% and 3.3% (23). In that study, the daily active user rate (median open rate) was only 4.0%. (23) These data highlight that the number of app installs has very little correlation with daily long-term usage.

Attrition and engagement rates (self-defined by study sponsors) in the pivotal studies for four FDA-authorized neuropsychiatric digital therapeutics are shown in Table 1. The studies reported significant benefits for the digital therapeutic vs. a control condition (Table 1), (10–15). Sample sizes were adequate, ranging from 170 to 1,149 participants (Table 1). Active intervention durations were relatively short ranging from 4 to 12-weeks (Table 1). Trial design, nature of therapy, incentives, and diagnosis influenced attrition. The Somyrst trials additionally reported 6 and 12-month follow up data. Attrition was lowest and compliance was highest in the pivotal study (14) of EndeavorRx for pediatric ADHD (Table 1) – this was a short 4-week trial of an interactive videogame where compliance was monitored electronically and there was close parental supervision. However, in their open 12-week study (15), the average missions engaged (with the videogame therapeutic) dropped by 34% at week 4 and by 50% at week 12 (Table 1). In the Somryst study for chronic insomnia (12), only 60% of subjects completed all 6 core modules of CBT and frequency of subject logins varied from 0 to 142 times (median of 25). While efficacy was sustained even at 12-month follow up, the decrease in insomnia score was greater in subjects who completed all 6 modules vs. those who did not. In the Somryst study for subclinical depression with insomnia (13), attrition rate was 58% at 6 weeks and on average only 3.5 of the 6 modules were completed. Patients completing <4 modules had no significant overall benefits vs. the control condition and were not different from the control condition at 6 months. In the reSET study for substance use disorder (10), the drop-out rate was low (12%) – this was likely because subjects were seen twice a week in the clinic, supervised by therapists, paid prizes ranging from “thank you” notes to up to $100 cash for compliance, and on average, earned $277 in prizes over 12 weeks. In their long-term follow-up (10), when this contingency incentive ended, the superiority of the digital therapeutic over the control condition also ended.

**Table 1:** Engagement and Attrition rates in the pivotal studies of FDA-cleared Digital Therapeutics for menta health.

**Reference number**	**N**	**Indication**	**Study intervention and dose**	**Trial design**	**Trial duration**	**Engagement**	**Attrition rate for active intervention arm**
(14)	348	Pediatric ADHD	RCT of Endeavor Rx vs. control video game 25 min per day, 5 days per week, for 4 weeks.	Hybrid	4 weeks	83%	6%
(12)	303	Chronic Insomnia	SHUTi (Somryst) six sequential modules completed within 9 weeks	Remote	9 weeks, with 12- month follow-up.	60.3%	9.2% 17.5%^a^
(15)	206	Pediatric ADHD	Open Label study of Endeavor Rx +/- pharmacotherapy 4 weeks (25 mins/day, 5 days/week), followed by a 4 week pause, and then another 4-week use of therapeutic.	Hybrid	12 weeks	68% (pharmacotherapy) 58% (no pharmacotherapy)^b^	12%(pharmacotherapy)12%(no pharmacotherapy)
(11)	170	Opioid Use Disorder	Therapeutic Education System (reSET-O) TES modules 3 times per week for 12 weeks.	In-Clinic	12 weeks	All sessions were supervised by a live therapist in the clinic.	20%
(10)	507	Substance Use Disorder	Therapeutic Education System (reSET) 4 TES modules per week for 12 weeks.	Hybrid	12 weeks	76.3%^c^	12%
(13)	1149	Subclinical depression and insomnia	SHUTi (Somryst) six sequential modules completed within 6 weeks	Remote	6 weeks, with 6 month follow up	58%	57% 61%^d^

a * Attrition at 12 month follow up. Studies used variable definitions and often did ot break down reasons*.

b * Compliance was the percentage of total possible recommended sessions. Engagement metrics were not reported in a standardized manner*.

c * 36.6 modules out of recommended 48 (range 0–72)*.

d * Attrition at 6 month follow up*.

### Closing the attrition-efficacy gap

Mental health conditions, like major depressive disorder, ADHD, and PTSD, require sustained treatment. Because the field of digital therapeutics is still in its early stages, currently, there is little long-term efficacy data. If even well-designed, gamified, digital therapeutics have a 50% drop in engagement in 3 months then the outlook for long-term efficacy is grim. While drop-out rates in clinical trials can be kept low through frequent clinician contact, gamification, feedback, and cash incentives, this is not practical in the real world and hence attrition rates will be far higher. Finally, if the costs of increasing engagement and compliance equals that of a getting live psychiatric care, then digital therapies would become less attractive as a scalable low-cost solution.

### Scientific gaps identified

Our scrutiny of published data also reveals several scientific gaps. First is the lack of standardized definitions of attrition and engagement in the field of digital therapeutics. Second is the lack of standardized reporting requirements by journals. A single digital therapeutic session can generate a dozen or more different metrics of how a user may interact with the app. Even widely used clinical trials reporting checklists, such as CONSORT, have not yet required the reporting of all such engagement metrics in digital trials. This makes it hard to extract such data from published reports and compare metrics across trials and products. Third, is the lack of a standardized definition of compliance. Fourth is the lack of standardized statistical methods, such as mixed models or last observation carried forward, to account for attrition and engagement biases in digital trials.

### Emerging solutions

Fortunately, several constructs are emerging as promising features to increase engagement – both related to external factors of motivation and UX design (24). Several factors such as ease of use, gamification, ability to personalize app, in-app symptom monitoring, numerical feedback, ability to chart progress, socialization within the app, and integration with clinical services, have been reported to increase engagement (17, 18). A machine learning analysis of 54,604 adult patients with depression and anxiety identified 5 distinct engagement patterns for digital cognitive behavior therapy over 14-weeks: low engagers [36.5% of sample], late engagers [21.4%], high engagers with rapid disengagement [25.5%], high engagers with moderate decrease [6.0%], and high persistent engagers [10.6%]. Depression improvement rates were lowest for the low engagers (22). This study suggested machine learning algorithms may be useful to tailor interventions and a human touch for each of these five groups. Kaiser Permanente found that integrating digital mental health solutions – provided *via* clinician referral – into their health care delivery system was able to successfully enhance engagement (25). Fears around privacy and data security for mental health data may be a factor in engagement and attrition for some participants and this should be addressed upfront. While we do not have all the solutions, encouraging the availability of raw data from clinical trials through trial registries, analyzing long-term real-world data on patient reported outcomes, user experience (engagement and compliance) and product reliability (18) will be important to enhance their utility.

Digital therapeutics for mental health are here to stay. As the pivotal studies demonstrate, they benefit a substantial number of patients. However, the gap between intention and real-world efficacy for digital therapeutics remains large. There is an urgent need to recognize this gap and for stakeholders–regulators, technology developers, clinicians, patients–to come together to close this gap and ensure that this form of treatment is useful to clinical populations.

## Author contributions

AN and PMD drafted the article. SB and MH provided critical edits. All authors helped with data interpretation of cited studies, contributed to the article, and approved the submitted version.

## Funding

The study was funded by a grant from the National Institute of Aging to PMD.

## Conflict of interest

MH has received grants from NIMH, NIA, Brain Initiative and Stanley Foundation for other projects. For other projects, PMD has received grants from NIA, DARPA, DOD, ONR, Salix, Avanir, Avid, Cure Alzheimer's Fund, Karen L. Wrenn Trust, Steve Aoki Foundation, and advisory fees from Apollo, Brain Forum, Clearview, Lumos, Neuroglee, Otsuka, Verily, Vitakey, Sermo, Lilly, Nutricia, and Transposon. PD is a co-inventor on patents for diagnosis or treatment of neuropsychiatric conditions and owns shares in biotechnology companies. The remaining authors declare that the research was conducted in the absence of any commercial or financial relationships that could be construed as a potential conflict of interest.

## Publisher's note

All claims expressed in this article are solely those of the authors and do not necessarily represent those of their affiliated organizations, or those of the publisher, the editors and the reviewers. Any product that may be evaluated in this article, or claim that may be made by its manufacturer, is not guaranteed or endorsed by the publisher.
